# BioBin: a bioinformatics tool for automating the binning of rare variants using publicly available biological knowledge

**DOI:** 10.1186/1755-8794-6-S2-S6

**Published:** 2013-05-07

**Authors:** Carrie B Moore, John R Wallace, Alex T Frase, Sarah A Pendergrass, Marylyn D Ritchie

**Affiliations:** 1 Center for Human Genetics Research, Vanderbilt University, Nashville, TN 37232, USA; 2 Center for Systems Genomics, Pennsylvania State University, University Park, PA 16802, USA

## Abstract

**Background:**

With the recent decreasing cost of genome sequence data, there has been increasing interest in rare variants and methods to detect their association to disease. We developed BioBin, a flexible collapsing method inspired by biological knowledge that can be used to automate the binning of low frequency variants for association testing. We also built the Library of Knowledge Integration (LOKI), a repository of data assembled from public databases, which contains resources such as: dbSNP and gene Entrez database information from the National Center for Biotechnology (NCBI), pathway information from Gene Ontology (GO), Protein families database (Pfam), Kyoto Encyclopedia of Genes and Genomes (KEGG), Reactome, NetPath - signal transduction pathways, Open Regulatory Annotation Database (ORegAnno), Biological General Repository for Interaction Datasets (BioGrid), Pharmacogenomics Knowledge Base (PharmGKB), Molecular INTeraction database (MINT), and evolutionary conserved regions (ECRs) from UCSC Genome Browser. The novelty of BioBin is access to comprehensive knowledge-guided multi-level binning. For example, bin boundaries can be formed using genomic locations from: functional regions, evolutionary conserved regions, genes, and/or pathways.

**Methods:**

We tested BioBin using simulated data and 1000 Genomes Project low coverage data to test our method with simulated causative variants and a pairwise comparison of rare variant (MAF < 0.03) burden differences between Yoruba individuals (YRI) and individuals of European descent (CEU). Lastly, we analyzed the NHLBI GO Exome Sequencing Project Kabuki dataset, a congenital disorder affecting multiple organs and often intellectual disability, contrasted with Complete Genomics data as controls.

**Results:**

The results from our simulation studies indicate type I error rate is controlled, however, power falls quickly for small sample sizes using variants with modest effect sizes. Using BioBin, we were able to find simulated variants in genes with less than 20 loci, but found the sensitivity to be much less in large bins. We also highlighted the scale of population stratification between two 1000 Genomes Project data, CEU and YRI populations. Lastly, we were able to apply BioBin to natural biological data from dbGaP and identify an interesting candidate gene for further study.

**Conclusions:**

We have established that BioBin will be a very practical and flexible tool to analyze sequence data and potentially uncover novel associations between low frequency variants and complex disease.

## Background

The era of successful Genome Wide Association Studies (GWAS) has contributed to over 1,596 published disease variant associations for over 249 traits (http://www.genome.gov/gwastudies/, accessed July 2012) Published GWAS results have increased the fields' understanding of heritable traits, highlighted novel disease associations that were critical for further biochemical and pharmaceutical development, and advanced the perception and understanding of genetic association and complexity of common diseases [1,2]. However, large proportions of variance in common complex diseases remain to be explained [3]. In the last few years, sequence technology has emerged as an successful approach to gain insight into other genetic components contributing to disease, including: low frequency or rare variants, mRNA transcript levels, etc.

In an effort to explain the additional heritability of complex traits, many researchers are investigating the effects of rare variants. The possible effects and complex heritatibility of rare variants are largely unknown; however, it is commonly believed that rare variants have bigger effect sizes (compared to common variants identified in GWAS) and can act alone, together with other rare variants, or jointly with common variants [2]. There is increasing evidence in published literature to support the hypothesis that rare variants contribute to the heritiability of common, complex disease. Recently, rare variants with moderate effect sizes have been associated with obesity, autism, schizophrenia, hypertriglyceridemia, hearing loss, complex I deficiency, age-related macular degeneration, kabuki syndrome, and type-1 diabetes [3-9]. The analysis of rare variants does not follow GWAS protocols. The sparseness of rare variants requires massive sample sizes for single-variant association tests. Combining rare variants together substantially reduces the sample size needed to find an association signal and also accounts for genetic heterogeneity.

Collapsing and/or binning methods have been popular because they reduce the degrees of freedom in the statistical test, are easily applied to case-control studies (not limited to family transmission filtering), can utilize whole-genome data, provide a means to enrich association signals by combining rare variants which are often otherwise undetectable, and allow for the investigation of collective polygenic inheritance. In the past five years, many collapsing methods have been published [5,10-18]. Most of these methods require significant bioinformatic pipeline infrastructure developed by the individual users to apply a method to their whole genome sequencing data, as they have been tested using simulations on the order of candidate gene studies. The limited regional approach used by most of these methods restricts novel discovery since a particular set of genes or pathways must be selected for analysis. In addition, considerable effort is necessary on the part of the user if the hypothesis warrants unusual or novel bin features. One method, VAAST has been designed to complete analyses using whole-genome sequence data. However, VAAST requires the user to generate and include a feature file, which contains the explicit boundaries for binning. This is trivial when the user wants to perform a rare variant burden test in genes, but much more complicated if the user needs to create pathway or regulatory region feature files.

The conceptual notion of binning is a very good one, but it is important to recognize four elements for improved algorithm development:

1. Complexity of interactions (epistatic and in aggregate) of rare and common variants

2. Potential non-independence between rare variants and between bins

3. Importance and possible limitation of "user" feature definition

4. Necessity of tool flexibility

Theoretically, there are many ways that variants can interact or act in aggregate to affect a phenotype; at this point, it is not known which will be true for the majority of common, complex disease. Variants in the same region can have opposing directions or effect, have no effect on the phenotype at all, or be part of complex epistatic interactions. Rare variants have been often considered to be independent of one another in method development, which is an assumption that is not necessarily true and should be considered in the analysis to protect from type I and type II errors [11,19]. Furthermore, bins, which contain rare variants, are not independent, particularly since the same variants can show up in multiple bins. Ideally, the simulations and statistical analysis used to develop, test, and support novel binning methods should reflect the possibility that variants and bins are not independent.

Users generate a hypothesis prior to performing an analysis, and in the case of collapsing algorithm; this includes choosing how bin boundaries are determined. Many current methods require additional input to inform the method of these boundaries, which makes testing multiple hypotheses or altering the hypothesis more difficult. Rather than requiring users to bin arbitrarily by RefSeq gene boundaries or select candidate gene regions, it is possible to bin within pathways, regions with known regulatory elements, evolutionary conserved sequences, and/or transcription factor binding sites. BioBin takes advantage of the power of prior knowledge, and the potential cumulative effect of rare variants in related biological networks.

Many current binning methods incorporate a fixed feature design focus for the statistical association test and take for granted that variants are collapsed in the most accurate and powerful way. The only exception is the application of functional prediction algorithms to filter the variants before binning to bin variants with similar directions of effect [11,19]. Adding a layer of specific filtering for the binning process allows the user to investigate biologically relevant collapsing boundaries that ultimately increase likelihood of discovering meaningful associations.

Our BioBin approach meets the criteria we have defined for improved binning algorithm development. Instead of focusing on a novel statistical test, we have concentrated on biology-driven automated bin generation. Based on the study hypothesis, the user selects binning features and BioBin creates appropriate feature level bins using information from one or more of the databases in our integrated database, call the Library of Knowledge Integration (LOKI). BioBin to can create bins based on many features, including: regulatory regions, evolutionary conserved regions, genes, and/or pathways. In addition, users can utilize complex binning, i.e. collapse only exons in pathways or perform regulatory and gene feature analyses simultaneously. The innovation of BioBin and incorporation of prior biological knowledge to automate bin generation allows the user the opportunity to test unique hypotheses [2].

Below, we present the methodology of BioBin, the underlying structure of LOKI that provides prior knowledge to BioBin, as well the results of our testing of BioBin with multiple datasets. We have tested BioBin using completely simulated rare-variant data, 1000 Genomes Project data, and NHLBI GO Exome Sequencing Project Kabuki dataset. Our tests show that BioBin is a flexible binning algorithm, useful for biological knowledge directed binning of rare variant data.

## Methods

### General framework

The goal of a low-frequency variant collapsing analysis is to compare the variant burden between two groups and identify bins with a significant excess of variants in one group compared to another. A bin analysis using BioBin follows these steps: 1) the user determines the feature to bin data (gene, pathway, etc), 2) BioBin executes the task of bin generation, 3) the user applies an appropriate statistical test to each bin. User-defined parameters and information from LOKI determine the boundaries of the bin. Users can adapt BioBin to their needs through the use of a configuration file, which can be used to adjust features or select certain database sources. When a variant is considered rare, which is defined as having a minor allele frequency less than the user-defined threshold in either the case or control group, it will contribute to the bin. By binning in this way, bins accumulate not only risk variants which have higher frequency in cases than controls but also potentially protective variants that have lower frequency in cases than controls. Considering the rarity of variants in the case and control groups separately reduces the number of false positive bins and the association between bin size and significance.

Additionally, the minor allele frequency threshold is configurable. This threshold determines the allele frequency limit under which variants are considered rare, and therefore binned. For example, if the threshold is set to 0.05, a locus with a minor allele frequency of 0.08 would not be included in a bin, but a locus with an allele frequency of 0.049 would be included. BioBin defines the minor allele at a given locus as the second most frequent allele in the target group. For a biallelic locus, this is always the rarer allele. For a polyallelic locus, the minor allele frequency considered in the binning process is calculated from the second most frequent allele, and all non-major alleles are binned identically. Common loci, defined to be those loci with allele frequencies above the binning threshold in both case and control groups, are not binned and are not considered in the analysis. Table 1 shows an example of major and minor allele frequency inclusion/exclusion from a single bin.

**Table 1:** Minor allele frequency threshold example

Major Allele (AF)	Minor Allele(s) (AF)	MAF	Variants Binned (Threshold = 0.05)
C: 0.97	T: 0.03	0.03	T
T: 0.80	A: 0.16, G: 0.04	0.16	
G: 0.95	C: 0.03, T: 0.02	0.03	C, T

Binning of major and minor allelle frequencies in sequence data given a minor allele frequency threshold of 0.05.

### BioBin software

BioBin is a C++ command line application that uses a prebuilt LOKI database. Source distributions are available for Unix-based operating systems (Mac OSX and Linux) and require minimal prerequisites to compile. The source distribution includes tools that allow the user to create and update the LOKI database by downloading the information directly from the respective sources. The computational requirements for BioBin are quite modest, a whole-genome analysis with 185 individuals took less than two hours. Since most current sequence studies are whole-exome, this analysis represents the most taxing use case in the foreseeable future The vast amount of data included in this particular analysis must be stored in memory, so the memory requirements can be high. For the same 185 individuals, BioBin required approximately 12 GB of memory. We have found that the primary driver of memory usage is the number of rare variants in the analysis, but with currently available sequence datasets, BioBin can be run quickly without access to expensive and specialized computer hardware [2].

### LOKI database

The utilization of prior biological knowledge is a powerful approach to inform collapsing feature boundaries. BioBin relies on the LOKI database, implemented in SQLite, for the integration of information from disparate data sources. Currently, LOKI contains information from sources such as: the National Center for Biotechnology (NCBI) dbSNP and gene Entrez database information [20], Kyoto Encyclopedia of Genes and Genomes (KEGG) [21], Reactome [22], Gene Ontology (GO) [23], Protein families database (Pfam) [24], NetPath - signal transduction pathways [25], Molecular INTeraction database (MINT) [26], Biological General Repository for Interaction Datasets (BioGrid) [27], Pharmacogenomics Knowledge Base (PharmGKB) [28], Open Regulatory Annotation Database (ORegAnno) [29], and evolutionary conserved region information from UCSC Genome Browser [30].

The creation of LOKI was a means to standardize interface and terminology between different sources that each contain potentially differing means of representing data. The three central concepts utilized in LOKI are *positions*, *regions *and *groups*. The term *position *is defined to be a single location in the genome, and could represent data such as rare variants (RVs), single nucleotide polymorphisms (SNPs), or single nucleotide variants (SNVs). A *region*, as defined by LOKI, refers to a segment with a defined start and stop position in the genome. Examples of regions include genes, copy number variants (CNVs), and evolutionary conserved regions (ECRs). LOKI uses the term *group *to refer to a collection of *regions *that are connected in some way. Each *source*, or database (such as those listed above), may represent the collection of *regions *in a different way, but LOKI standardizes all of the information, which allows for uniform access and usage of the prior biological knowledge [2].

SQLite is the relational database management software chosen for the implementation of LOKI because it does not require a dedicated database server. Before using BioBin, the user must run the provided installer scripts to download and process the biological knowledge into a single database file (~ 7 GB range). Due to the amount of information downloaded from the various sources, a system building a LOKI database should have at least 50 GB of disk storage available. When used by BioBin, LOKI runs locally and needs no connection to the Internet [2].

### Binning approach

**Figure 1:**
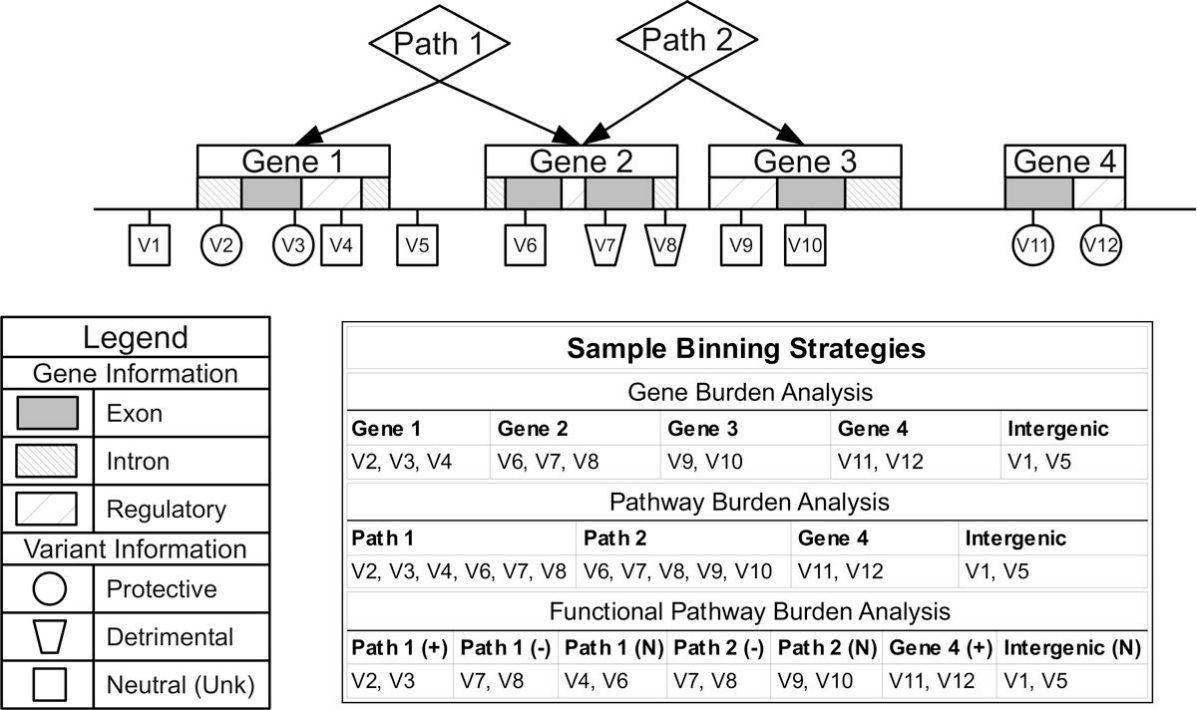
**Example binning strategies**. Binning example using four genes, two pathways, and functional prediction.

Due to ease of access and clearly defined database schema, we chose NCBI dbSNP and NCBI Entrez Gene as our authoritative sources of position and regional information. Pathway/group bins were created using sources in LOKI (detailed in software section). Inter-region bins of a user-specified size were generated by BioBin to catch variants that did not fit into the user-defined features. Therefore, after BioBin feature selection, inter-region bins were created. In other published collapsing methods, intergenic variants would typically not be included in the analysis. Common binning strategies are shown in Figure 1, which include gene, pathway, and intergenic features, and the possibility of applying a functional prediction filter.

### Statistical testing

**Figure 2:**
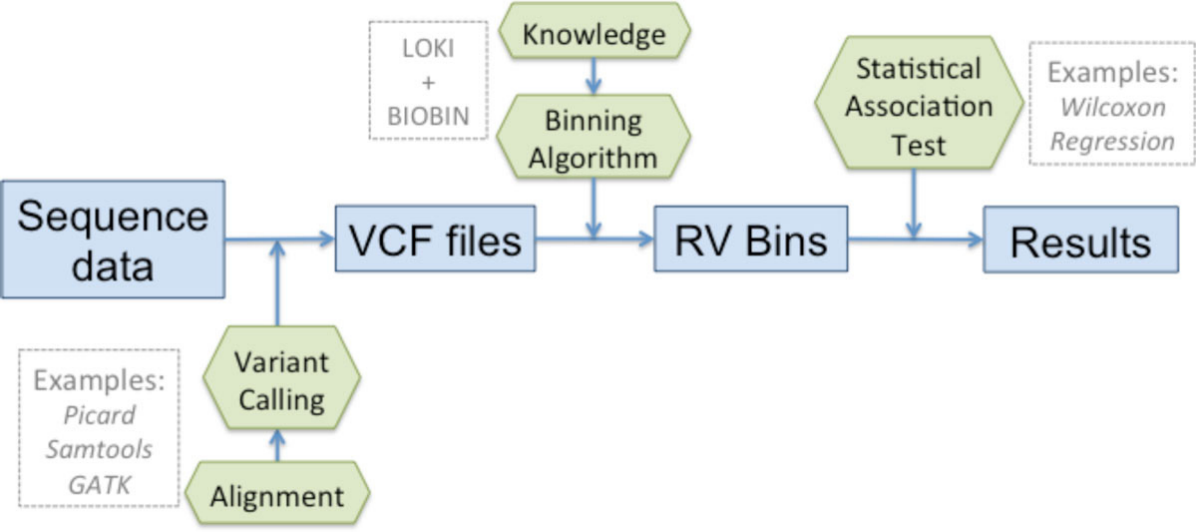
**Pipeline for BioBin analysis**. Pipeline for BioBin analysis. Blue squares correspond to data, green hexagons correspond to bioinformatic or statistical method applications.

BioBin is a tool to create new feature sets that can be examined in subsequent statistical analyses. BioBin output provides information about the bins generated, summary values, and a matrix of variant sums per individual for each bin generated. Care should be taken with bin output to choose a statistical test appropriate to the hypothesis being tested, the question of interest, and the type of data tested. The innovation of BioBin is its capacity to employ feature selection using a knowledge base (see Figure 2). The resulting RV bins can be used in a variety of statistical analyses; there are explicit situations that require the use of regression analysis (logistic, linear, or polytomous), Fisher's exact test, or permutation of unique statistical test, etc. In order to appeal to the broadest user base, no specific statistical test is implemented into BioBin. In comparison, other collapsing methods do not generate bins based on biological knowledge; instead, they focus on the association test and results after bin generation [2]. The results we present below were calculated using a Wilcoxon 2-sample rank sum test implemented in the R statistical package [31].

### Simulation strategy

To test BioBin, we simulated genetic data using SimRare [32]. SimRare is a GUI interface simulation program built on top of a forward time simulator, simuPOP [33]. simuPOP simulates the introduction and evolution of rare variants and allows complex fitness and selection modeling [http://simupop.sourceforge.net, https://code.google.com/p/simrare/]. There are a few reasons SimRare is preferable to simuPOP for the end user: it is computationally efficient, time efficient, and reduces the number of linking scripts needed to create a many replicates of simulated data.

We used an additive multi-locus selection model with a selection distribution described by Kryukov [34]. The mutation rate was set at of 1.8 × 10^-8 ^per nucleotide per generation. The population sizes were N_e _= 8100, 8100, 7900, and 900000 with 5000 generations, 10 generations, and 370 generations respectively. A fixed 5kb region was simulated using 250 replicates of the same evolution parameters. In this instance the term replicate is a realization of the forward-time simulation using the given input parameters, and each replicate is not identical since evolution cannot be exactly repeated due to randomness. However, more replicates slightly increase the diversity of the final simulated data.

#### Assessing type I error

To evaluate type I error, we generated a sample data set with the following parameters: 1.0 odds ratio for protective mutations, 1.0 odds ratio for detrimental mutations, and an additive mode of inheritance. We did not incorporate missingness or unphenotyped individuals. The type I error was computed as the percentage of the 4,000 replicates with a p-value <= 0.05. A type I error rate above 5% would indicate a high likelihood of false positives and a type I error rate lower than 5% would indicate a conservative test.

#### Power

To evaluate power, we generated a sample data set with the following parameters: 0.9 odds ratio for protective mutations, 2.5 odds ratio for detrimental mutations, and an additive mode of inheritance. Again, we did not incorporate missingness or unphenotyped individuals. The power was computed as the percentage of the 4,000 replicates with a p-value <= 0.05.

### 1000 Genomes Project data comparison

Simulation studies are useful for many reasons but cannot account for 100% of the heterogeneity in natural data. Low frequency variants are often specific to continental populations or even to individual populations [35]. 1000 Genomes Project data was used to test BioBin, and contrast results with our completely simulated rare-variant data. The 1000 Genomes Project was started in 2008 with the aim to deliver deep characterization of variation in the human genome. As of October 2011, the sequencing project included sequence data for 1094 individuals, with the intent to sequence 2,500 individuals by the time of the project's completion. The project reports capture 95% of all variation with frequencies greater than 1% [36]. We utilized the 1000 Genomes Project data in two ways, 1) adding resequenced variants to a study of cases and controls from 1000 Genomes data of the same ancestral population to determine BioBin's ability to resolve a large simulated signal in the midst of various amounts of background variation 2) pairwise rare variant burden comparison using 1000 Genomes Project data between two ancestral populations to expose known patterns of population stratification.

#### 1) Adding resequenced variants

First, we tested BioBin by adding a simulated variant with 100% penetrance to two genes on chromosome 7 in a case-control sample composed of individuals of European descent (CEU). Individuals were randomly assigned case/control status from the 87 unrelated CEU samples. As the added variant had 100% penetrance, each case was assigned as a heterozygote and each control was homozygous referent for the simulated variant. An additive genetic model was used to detect rare variant burden under three minor allele frequency bin threshold conditions (0.02, 0.05, 0.10). Testing the bins under different allele frequency threshold conditions affords an easy way to test the same bins with more or less variants, which can provide some information about the sensitivity of this binning method.

We added our simulated variant to the gene *GLI3 *(GLI family zinc finger 3), a large gene associated with polydactyly syndrome [OMIM 174200]. This gene has between 262 and 602 variant loci indicating a very polymorphic gene in the CEU genome (including the simulated variant). The addition of one simulated variant loci added a total of 43 variants (each case is heterozygous and only contributes one variant to bin). In a second test, we randomized the case/control status again and added a simulated variant to the gene *CAV2 *(protein cavelolin-2, induced during adipocyte differentiation), with between 9-20 variant loci (including the simulated variant). BioBin was then used for a rare variant burden test across the chromosome 7 genomic region to see if either the *GLI3 *or *CAV2 *signal could be identified as significantly associated using a Wilcoxon Rank Sum test.

#### 2) Pairwise comparison in YRI and CEU

Second, we compared two populations, YRI and CEU, from 1000 Genomes Project to highlight rare variant burden differences. Rare variants found at different frequencies among ethnic-specific populations and sequence data is available through 1000 Genomes Project for a large number of individuals (October 2011 release ftp://ftp-trace.ncbi.nih.gov/1000genomes/ftp/release/20110521/). This analysis included 87 CEU samples and 88 YRI samples. We used BioBin to conduct a pairwise comparison of rare variant burden differences (MAF < 0.03) based on known gene regions (start and stop positions form bin boundaries), intergenic regions (intergenic variants caught by 50kb bins) and known pathways (gene bins in the same pathway are collapsed into one pathway bin).

### NHLBI Kabuki dataset test

We utilized the NHLBI Kabuki dataset available on dbGaP (http://www.ncbi.nlm.nih.gov/gap/) April 2012. According to the authors of the original study, ten unrelated individuals with Kabuki syndrome were sequenced: 7 of European ancestry, 2 of Hispanic ancestry, and one of mixed European and Haitian ancestry. Shotgun fragment libraries were hybridized to custom microarrays, and then enriched using massively parallel sequencing [8]. The raw fastq files were downloaded from dbGaP and processed using standard exome algorithms: bwa, samtools, picard, GATK, and bedtools. We used publically available Complete Genomics whole-genomes sequences for 54 unrelated individuals from 11 populations as the control group for this experiment [37]. We used a MAF binning threshold of 0.05 and filtered out variants present in 1000 Genomes Project data to collapse rare variants based on known gene regions (start and stop positions form bin boundaries) and known pathways (gene bins in the same pathway are collapsed into one pathway bin).

Although a sample of ten individuals across multiple ancestries do not provide reasonable power to achieve statistical significance for identified rare variant trends, it was still useful for showing how BioBin can be used to prioritize bins based on rare variant burden differences.

## Results

### Simulation results

Using SimRare, a 5kb genomic region was simulated with an average of 968 rare variants as described in the methods section for four sample sizes (N = 2000, 1000, 500, 250). Case/control status was evenly and randomly assigned in each of the 4000 replicates. The type I error rate was calculated as the number of replicates with p-value <= 0.05 divided by the total number of replicates. Shown in Table 2, the type I error tests indicate that the Wilcoxon 2-sample rank sum test was marginally anticonservative. This conveys that using a Wilcoxon 2-sample rank sum test controls the false-positive rate in BioBin. Similarly, power was assessed using a 0.9 odds ratio for protective mutations and 2.5 odds ratio for detrimental mutations with an additive mode of inheritance. We calculated the power of the Wilcoxon test as the proportion of the 4,000 replicates at each sample size with a p-value <= 0.05. The power dropped to less than 80% in sample sizes less than 1000.

**Table 2:** Simulation results

Sample Size	Type I Error Rate	Power
2000	0.05375	0.991
1000	0.0495	0.934
500	0.055	0.7575
250	0.0545	0.503

Rare variant simulation using SimRare to assess power and type I error rate.

### 1000 Genomes comparisons

Statistical analysis using bin output can be complicated considering dependent bin and variant architecture, variance in bin size, and complex genetic models. We used 1000 Genomes CEU data to evaluate if a) BioBin could detect bins containing a 100% penetrant simulated variant b) determine if bin size (number of variants in a bin) could affect the strength of a simulated association signal by seeding the variant into two genes with very different numbers of non-simulated variants, testing different minor allele frequency binning thresholds to see how the p-value changes with respect to small changes in the number of variants collapsed in the bin. BioBin can detect bins containing the penetrant simulated variant, but this is affected greatly by the number of other variants in a given bin. The results are shown in Table 3: the MAF threshold corresponds to the binning threshold, the number of loci indicates the number of physical positions in the bin region that contribute at least one variant, the number of bins corresponds to the number of bins generated on chromosome 7. Table 3 also shows the number of significant bins for each analysis and the rank of the gene of interest. For example, as shown in Table 3, the signal was completely mitigated by noise in the *GLI3 *bins. *GLI3 *has a large number of variants in addition to the simulated variant. While the statistical test did not identify *GLI3*, there were also no false positives. There were no significant bins in any of the three *GLI3 *analyses. The *CAV2 *bin was statistically significant after Bonferroni correction when the minor allele frequency threshold was less than or equal to 0.05, a gene with a very low number of additional non-simulated variants.

**Table 3:** Analysis with simulated variants in *GLI3 *and *CAV2*

Gene	MAF Thres	Location	Size	Loci	# Bins	# Sig. Bins	Overall Rank	Unadj. P-val*	Adj. P-val
*GLI3*	0.02	7:42000547-42276618	276kb	262	1539	0	1377	0.9828	1
*GLI3*	0.05			431	1692	0	1288	0.7723	1
*GLI3*	0.10			602	1730	0	1273	0.7404	1

*CAV2*	0.02	7:116139654-116148595	109kb	9	1612	1	1	1.731e^-17^	2.791e^-14^
*CAV2*	0.05			11	1701	1	1	2.338e^-14^	3.976e^-1^
*CAV2*	0.10			20	1735	0	3	0.0005869	1

A simulated variant was added to two genes, *GLI3 *and *CAV2*. The genes were used to test signal mitigation in the presence of a large versus small number of variants in the bin. The adjusted p-values were calculated using a Bonferroni correction to account for the total number of bins generated.

These two simulated variants scenarios were tested separately in BioBin. As a result, the phenotypes were randomly assigned, thus allowing for different bins to be generated based on which and how many variants met the MAF binning threshold criteria.

**Figure 3:**
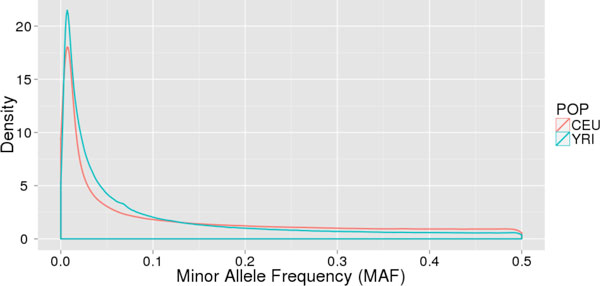
**Minor allele frequency distribution for CEU and YRI**. Minor allele frequency distribution for CEU (red) and YRI (blue) populations. The African population has a higher density of low frequency variants.

We also tested BioBin using whole-genome population data from 1000 Genomes Project. Because of known population stratification between CEU and YRI, this is a reasonable rare variant burden test. using whole-genome data. For 87 CEU and 88 YRI individuals, there are approximately 11 million variants (rare and common) in the CEU sample and approximately 18 million variants in the YRI sample, an indication of similarity/dissimilarity to the reference genome In addition to having more variants, it is also clear that of these variants, there is a larger proportion of low frequency variants in the YRI sample. Figure 3 shows the whole-genome minor allele frequency density distribution of CEU and YRI populations.

Using a MAF binning threshold of 0.03, we binned genes, pathways, and intergenic regions. The results of this test are shown in Table 4, where "Feature Type" defines the bin boundaries, "Bins" corresponds to the number of bins generated by prior knowledge in BioBin given the feature type, "Sig. Bins" is the number of bins (out of the total in that feature type) that were statistically significant after a Bonferroni multiple testing correction, and "% Sig." is the proportion of significant bins divided by the total number of bins. However, because of evolutionary pressures we expected less diversity in gene regions than intergenic regions because it is likely coding regions have evolved lower mutation rates than noncoding regions. Pathways show the highest proportion of significant bins, however, significantly different but common gene regions are likely influencing the pathway analysis. In all three features, there is a surprisingly high proportion of significant bins.

**Table 4:** Feature analyses applied to CEU-YRI comparison

Feature Type	Bins	Sig. Bins	% Sig
Genes	31519	17436	55.3190
Intergenic	40362	28447	70.4797
Pathways	118221	99445	84.1179

Four features were tested on the CEU-YRI population comparison using BioBin to investigate differences in rare variant burden. The table shows the total number of bins, number of signficant bins (after Bonferroni correction), and the percent of significant bins

### NHLBI Kabuki dataset

We used BioBin to collapse the whole-exome data for 10 Kabuki individuals with 54 individuals from Complete Genomics whole-genome data. The 10 Kabuki cases were sequenced using an exome-capture kit for an average coverage of 40x on the mappable, targeted exome [8]. The 54 Complete Genomics samples were sequenced with an average genome-wide coverage of 80X [37]. In the original Kabuki analysis, Ng et al. used a filtering method to identify *MLL2 *as a possible causative gene for Kabuki syndrome. In this analysis, in order to compare the cases and controls, we filtered both datasets by exome boundaries (available from UCSC) and filtered out variants present in the 1000 Genomes Project Phase I data. BioBin produced the MLL2 gene bin with 125 total variant loci (184 total variants) at a minor allele frequency threshold of 0.05, but was not significant (p-value = 0.4718).

## Discussion

### Simulation results

Shown in Table 2, the Wilcoxon 2-sample rank sum test is slightly anticonservative but independent of sample size and is not of much concern. Further exploration should be performed to evaluate the relationship between rare variant allele frequency distribution and type I error, to calculate the type I error using a variety of sample population sizes, and to examine if the number of variants in a bin consistently inflate the false positive rate. The power is greater than 90% at sample sizes of 1000 and 2000 individuals. The power drops to 75% at a sample size of 500 and 50% at a sample size of 250. While the power drops dramatically with decreasing sample size, it is important to note that power to detect associations relies heavily on the effect size of the variants [38]. In the case of population stratification, the penetrance is quite high and the effect size is related to the allele frequency difference between the two populations, which is often several fold higher than the 2.5 odds ratio of the simulated dataset.

### 1000 Genomes Project comparison

1000 Genomes Project has provided a unique opportunity to evaluate bins on natural data. In the first test, BioBin was used to identify bin containing simulated variants with known association to a simulated phenotype. These steps were taken for two genes with very different rare variant backgrounds. For example, using a minor allele frequency-binning threshold of 0.05, *GLI3 *had 431 variant loci with 1099 total variants in the CEU group. At the same binning threshold, *CAV2 *had 11 variant loci with 57 variants in the CEU group. We expect causative rare variants to be highly penetrant, but clearly the power of BioBin to detect a signal with a large main effect is greatly affected by the number of other variants in the bin. Using *GLI3 *as an example, when there were greater than 300 variants in the bin, the true signal was mitigated by the noise. However, when there were less than 20 variants in the bin, a single variant with a large main effect was easily detected. Optimistically, in natural data each case has one or more rare-variants in addition to the natural background variation (which would also be present in controls). The number of variants in the background variation is deterministic of detecting a signal; this exemplifies the importance of maintaining bins of a reasonable size. However, if there are more causative variants acting in concert with a relatively high penetrance, this signal will be more robust. It also emphasizes the impact of flexible binning. The ideal bin will have as many rare variants with the same direction of effect and very few neutral variants. This has been shown to improve power in other methods [11]. To do this, other available methods would require some bioinformatic filtering in addition to the collapsing method. For BioBin, this is a matter of choosing more explicit binning instructions. For example, split pathway "A" bin into pathway "A" exons and pathway "A" introns. Another way to reduce the number of variants in a bin is to apply functional predictions. One could use Polyphen output for variants in the input dataset, and BioBin with generate pathway "A" risk variants and pathway "A" non- risk variants. One potential disadvantage of Polyphen or any other prediction algorithm is that only variants with prediction scores would be included in the bins, while all other would be excluded from the analysis. To accommodate the needs of the user, BioBin accepts a generic form of prediction output, which makes it possible to use almost any available tool but also accommodates novel functional prediction algorithms.

In the subsequent test using 1000 Genomes Project whole-genome data, we used BioBin to identify features with significant differences in rare variant burden. A similar method was used by Madsen and Browning and Moore et. al [2,12]. A population-genetics approach implies case/control status by ancestry identification, but also retains natural qualities of data. Madsen et al. used five 100kb regions from Encode III data sequenced in different populations. To mimic disease resequencing, they grouped exonic rare variants by each region and compared between African Yoruba individuals (YRI) and European descent individuals (CEU) [12]. Similarly, we compared three feature types between two ancestral populations from 1000 Genomes Project. This is interesting because it provides another approach to test BioBin, but also because population stratification is a known issue in genomic studies and could be useful knowledge for more traditional case/control analyses in sequence data. BioBin discovered features with rare variant burden differences between CEU and YRI populations. For each feature tested (genes, intergenic, and pathways), the majority of bins had statistically significant differences (adjusted using Bonferroni multiple testing correction) in rare variant burden. We investigated allele frequencies in the binned loci in CEU and YRI individuals, over 65% of the variant loci were fixed in the CEU individuals. This is not surprising since it is well known that individuals of African descent have more variation than individuals of other ancestral groups when compared to the reference genome. The difference in rare variation is driving the high percentages seen in Table 4.

It is also interesting that the rate of variation between CEU and YRI is not consistent across each feature tested. Gene bins had fewer significant rare variant differences than intergenic and pathway bins. Perhaps we see overall less variability in these regions because mutations are less tolerated. It has been suggested that genes undergo adaptive evolution, thus regions with potential for highly deleterious mutations such as genes, evolve lower mutation rates [39,40]. This stratification is an interesting result for BioBin but also indicates a need to further investigate the possible effect of feature specific rare variant stratification in sequencing studies.

### NHLBI Kabuki dataset

Ng et al. filtered out 1000 Genome variants and other non-causative variants from previous Kabuki studies. They also considered only nonsynonymous variants with predicted function. However, ten individuals with whole-exome data is not a large enough case sample size for sufficient power in a Wilcoxon 2-sample rank sum test. The uneven sample size and different sequencing approach for cases and controls is major limitation. To compare the cases and controls, we filtered both datasets by exome boundaries (available from UCSC). Because of the sample size limitation, reducing potentially neutral variants that contribute noise was advantageous. We removed any variants that were present in the 1000 Genomes Project Phase 1 release (14 populations, 1094 individuals) [36]. Then we used a minor allele frequency- binning threshold of 0.05 to bin gene and pathway features.

We have not used the same exact filters and have very low power for a case-control study, which together, likely explain why *MLL2 *was not more significant in this analysis. In addition, population stratification exists in the cases and controls and was not accounted for in this analysis. Lastly, *MLL2 *has 54 exons and quite a bit of neutral variation. As shown in Table 3, larger bins with increased background variation make causative signals harder to detect. While replication for *MLL2 *was absent, one of the top pathways included *EMG1*, previously associated with Bowen- Conradi syndrome (pathway adjusted p-value < 0.001, gene adjusted p-value < 0.001). Bowen-Conradi syndrome has a much more severe phenotype, but shares two disease characterizations with Kabuki: impaired growth and mental retardation [41]. Given the described analysis, BioBin results should be utilized as a prioritization method; thus, it would require a much larger sample size to investigate the robustness of the *EMG1 *association. To improve this analysis, one could potentially use a principle components analysis to adjust for the variant confounding between the two groups in a regression analysis or perform a permutation test to help adjust for unknown confounding. A better test data set would include at least 150-200 individuals that were sequenced on the same platform. Overall, BioBin can be used as a filtering mechanism to group data and evaluate rare variant burdens between two groups, but requires a more substantial sample size to gain power to detect significance.

## Conclusions

To explain more phenotypic variation in common complex disease, it is imperative to consider genetic variation beyond common single nucleotide polymorphisms. Rare variant analyses are appealing since effect sizes are likely larger [1]. However, to improve power, one must consider groups of rare variants with similar properties. BioBin is a novel collapsing method that uses allele frequency data and biological information to bin rare variants. There are many advantages of using a biologically informed method [2]:

1. Capability for whole-genome and whole-exome analyses

2. Practical method for data reduction in sequence data analysis

3. Utilizes domain knowledge to prioritize results for association testing

4. Accurate binning increases the statistical power to detect associations

5. Gene based output can formatted to identify GxG, GxE, and gene-drug interactions

6. Can be combined with common variant methods

7. Provides framework to integrate numerous types of complex data sets

BioBin is unique because it relies on LOKI and does not include any statistical method. The user can easily test complicated and interesting hypotheses on many features. LOKI provides access to integrated biological knowledge (pathways, groups, interactions, ECRs, regulatory regions, etc.), which is valuable to researchers that do not want to spend considerable effort to combine this knowledge manually. Additionally, the output of BioBin can be subsequently analyzed using the association tests most appropriate for their data analysis. For any given bin, many statistical tests from other published collapsing methods can be applied to BioBin output.

Using a Wilcoxon 2-sample rank sum test, we have shown that the type I error rate is controlled, but that substantial (>500) sample sizes are needed to have greater than 80% power to detect rare variants with modest effect sizes. With BioBin, we were able to find simulated variants in genes with less than 20 loci, but found the sensitivity to be much less in large bins. This will be investigated further so that we can better control bin size for gene regions or groups, such as pathways. From the population comparison analysis, we learned more about the large degree of population stratification between CEU and YRI populations from 1000 Genomes data. Lastly, we were able to apply BioBin to natural biological data from dbGaP.

With the rapid increase in available sequence data and available biological knowledge, we believe that BioBin will continue to be a useful analysis tool. The ability to quickly form and test unique, interesting, and biologically relevant hypotheses using aggregated low frequency variation will aide scientists in revealing hidden heritability for common complex disease. Software for BioBin is publicly available for download at https://ritchielab.psu.edu/ritchielab/software.

## Competing interests

The authors declare that they have no competing interests.

## Authors' contributions

CBM carried out the analyses, performed the sequence alignment and drafted the manuscript. JRW wrote and maintains BioBin code. ATF wrote and maintains LOKI. SAP participates in the design of LOKI and helped with manuscript revisions. MDR participated in the design of the study and manuscript revisions. All authors read and approved the final manuscript.

## Declarations

The corresponding author funded the publication costs for this article.

This article has been published as part of *BMC Medical Genomics *Volume 6 Supplement 2, 2013: Selected articles from the Second Annual Translational Bioinformatics Conference (TBC 2012). The full contents of the supplement are available online at http://www.biomedcentral.com/bmcmedgenomics/supplements/6/S2.
